# Modeling and mapping the burden of disease in Kenya

**DOI:** 10.1038/s41598-018-28266-4

**Published:** 2018-06-29

**Authors:** Michael Frings, Tobia Lakes, Daniel Müller, M. M. H. Khan, Michael Epprecht, Samuel Kipruto, Sandro Galea, Oliver Gruebner

**Affiliations:** 1 Humboldt-Universität zu Berlin, Geography Department, Berlin, Germany; 2 Leibniz Institute of Agricultural Development in Transition Economies (IAMO), Halle (Saale), Germany; 3 University of Bielefeld, School of Public Health, Department of Public Health Medicine, Bielefeld, Germany; 4 University of Bern, Center for Development and Environment (CDE), Bern, Switzerland; 5 Kenya National Bureau of Statistics, Nairobi, Kenya; 6 Boston University, Department of Epidemiology, Boston, MA USA; 7 University of Zürich, Epidemiology, Biostatistics, and Prevention Institute (EBPI), Zürich, Switzerland

## Abstract

Precision public health approaches are crucial for targeting health policies to regions most affected by disease. We present the first sub-national and spatially explicit burden of disease study in Africa. We used a cross-sectional study design and assessed data from the Kenya population and housing census of 2009 for calculating YLLs (years of life lost) due to premature mortality at the division level (N = 612). We conducted spatial autocorrelation analysis to identify spatial clusters of YLLs and applied boosted regression trees to find statistical associations between locational risk factors and YLLs. We found statistically significant spatial clusters of high numbers of YLLs at the division level in western, northwestern, and northeastern areas of Kenya. Ethnicity and household crowding were the most important and significant risk factors for YLL. Further positive and significantly associated variables were malaria endemicity, northern geographic location, and higher YLL in neighboring divisions. In contrast, higher rates of married people and more precipitation in a division were significantly associated with less YLL. We provide an evidence base and a transferable approach that can guide health policy and intervention in sub-national regions afflicted by disease burden in Kenya and other areas of comparable settings.

## Introduction

The Global Burden of Disease (GBD) study provides an excellent framework to quantify the magnitude of health loss due to diseases, injuries, and risk factors^1^. GBD studies quantify health loss through both mortality and morbidity by using the so-called disability-adjusted life years (DALYs)^2,3^. In Kenya for example, 78.3% of total DALYs are constituted by years of live lost (YLL) due to premature mortality^4^, with the leading causes HIV/AIDS, lower respiratory infections, diarrheal diseases, tuberculosis, and malaria^5^.

However, most of the GBD studies have focused on the national level^6^, missing potentially significant variations at the sub-national level^7^. Although some studies assessed disease burden at the sub-national level^8–11^, only a few considered spatial patterns in these measures^12,13^. Knowledge about sub-national regions that exhibit significant above or below average disease burden is of particular interest for deciding where to intervene to improve population health.

Yet, there remains much we do not know about the sub-national distributions of risk factors of disease burden so that we have limited knowledge about where health interventions will be most efficient. Many low and middle-income countries lack disaggregated health statistics that are needed for sub-national studies and spatial analyses. A possibility to overcome this issue may be census data that is more often available and could be useful to analyze disease burden due to e.g. premature mortality.

To the best of our knowledge, no study systematically assessed the spatial patterns of disease burden due to premature mortality with sub-national data across an entire country in Africa. One notable exception however, is provided by Manda and Abdelatif^14^, who analyzed the spatial-temporal variation of mortality risk across South African municipalities. However, they did not account for important risk (e.g., infectious disease), environmental or socio-demographic factors (e.g., climate, ethnicity). Furthermore, their study did not explicitly assess spatial clusters of life years lost.

We set out to investigate the spatial distribution of disease burden based on the most recent population and housing census 2009 in Kenya. Specifically, we aimed to 1) detect spatial clusters of YLLs at the division level (n = 612), and to 2) identify variables that are associated with the YLLs at this level.

## Results

**Figure 1:**
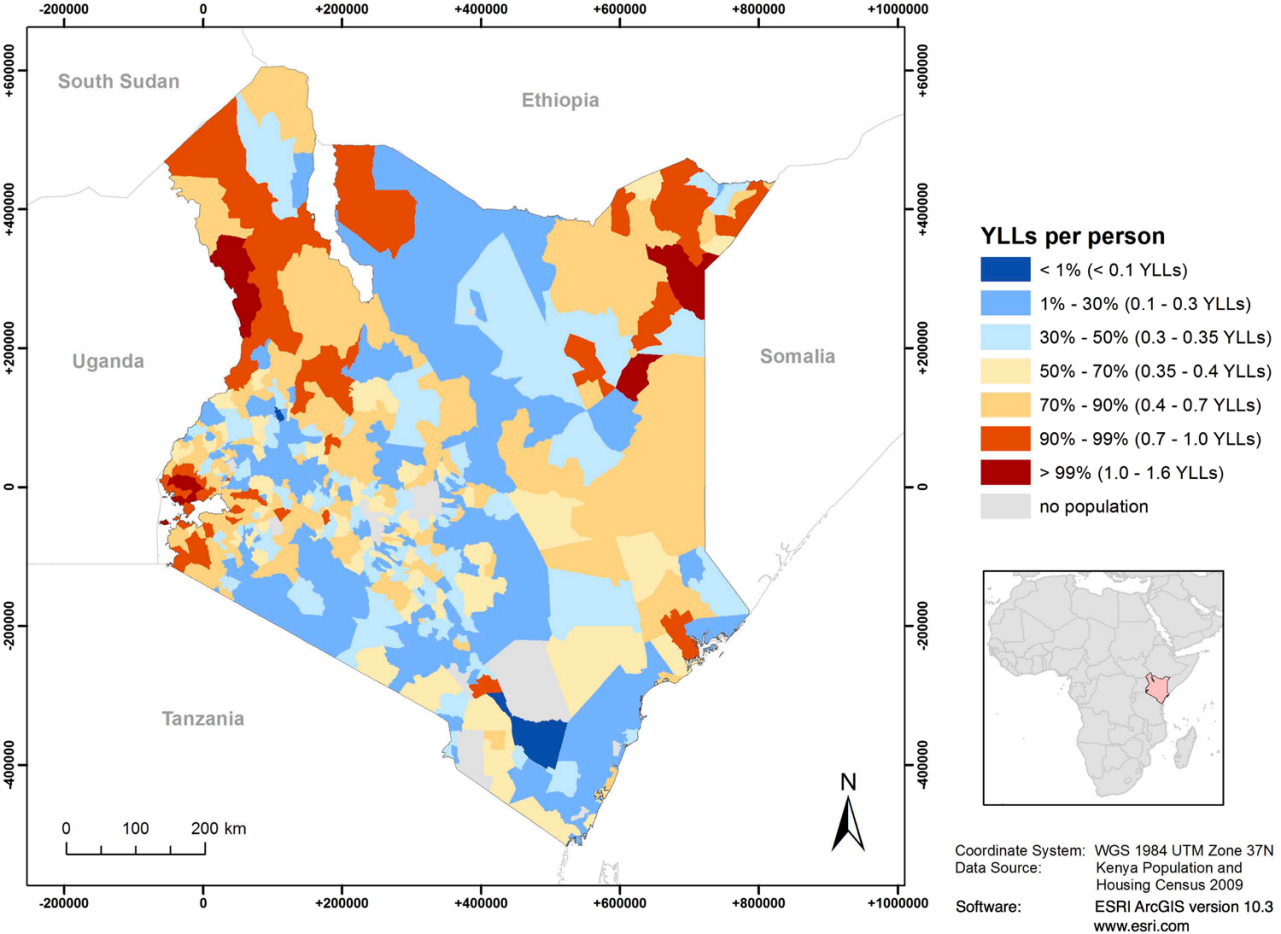
Years of life lost (YLL) due to premature death at the division level in 2009.

**Figure 2:**
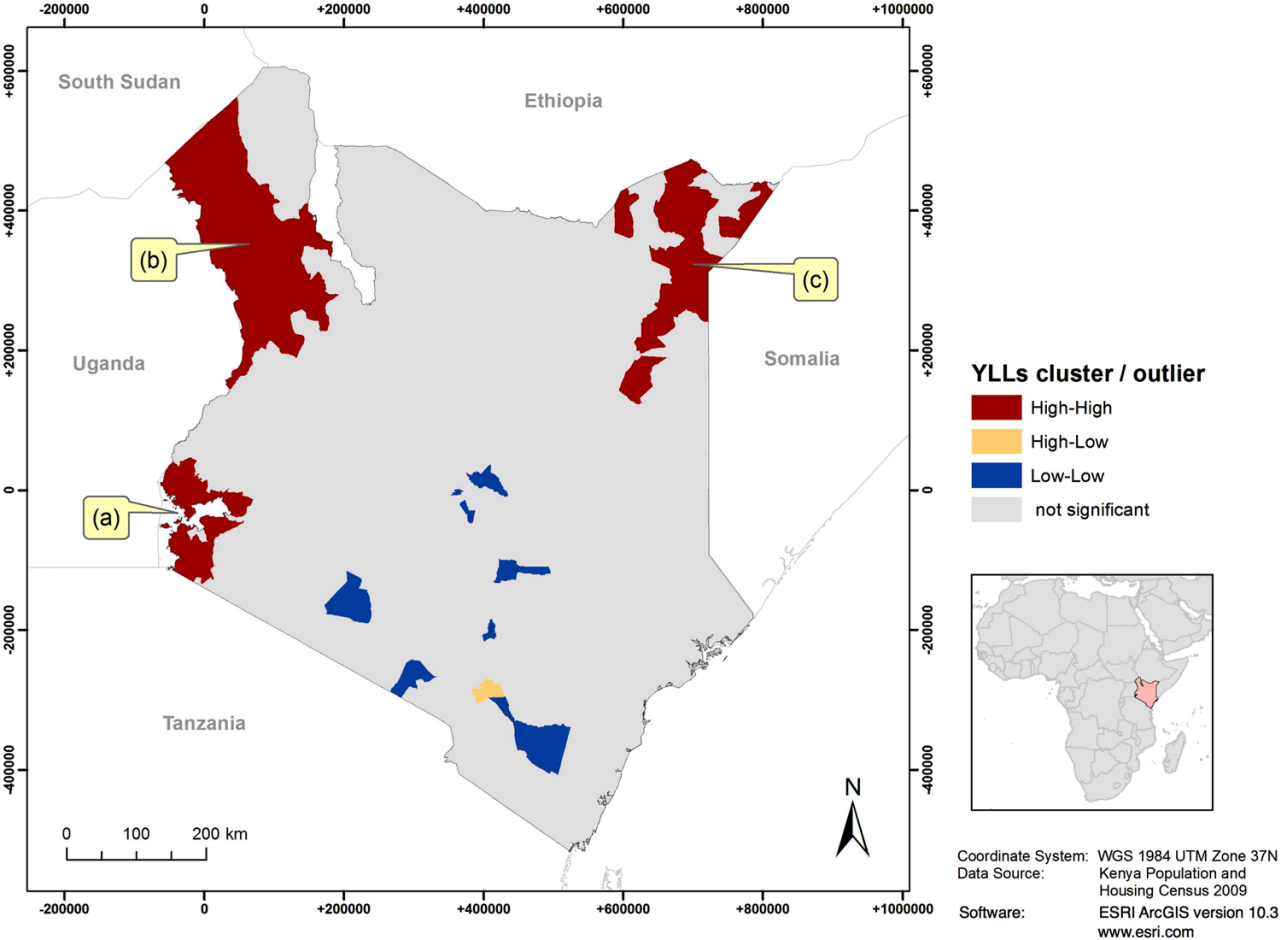
Significant spatial clusters of years of life lost (YLLs) per person at the division level. The map shows three clusters of divisions in which high values of YLL (above average) were found next to each other, one near Lake Victoria (**a**) one in Turkana County (**b**) and one in the border triangle with Ethiopia and Somalia (**c**).

We noted that YLL exhibited a distinct geographic pattern, with higher YLLs in western, northwestern, and northeastern Kenya (Fig. 1). We found small but significant spatial clustering of YLL across Kenyan divisions (global Moran’s I = 0.20, p-value < 0.001). Figure 2 shows significant (p-value < 0.001) local spatial clusters of high YLL rates (a) near Lake Victoria in western Kenya, (b) in Turkana County in the northwest, and (c) near the border with Ethiopia and Somalia in the northeast. Significant spatial clusters of low YLLs were found in central and southern Kenya. Figures 1 and 2 of the supplementary file contain additional information for YLL based on the Kenya specific life expectancy (not reported).

**Figure 3:**
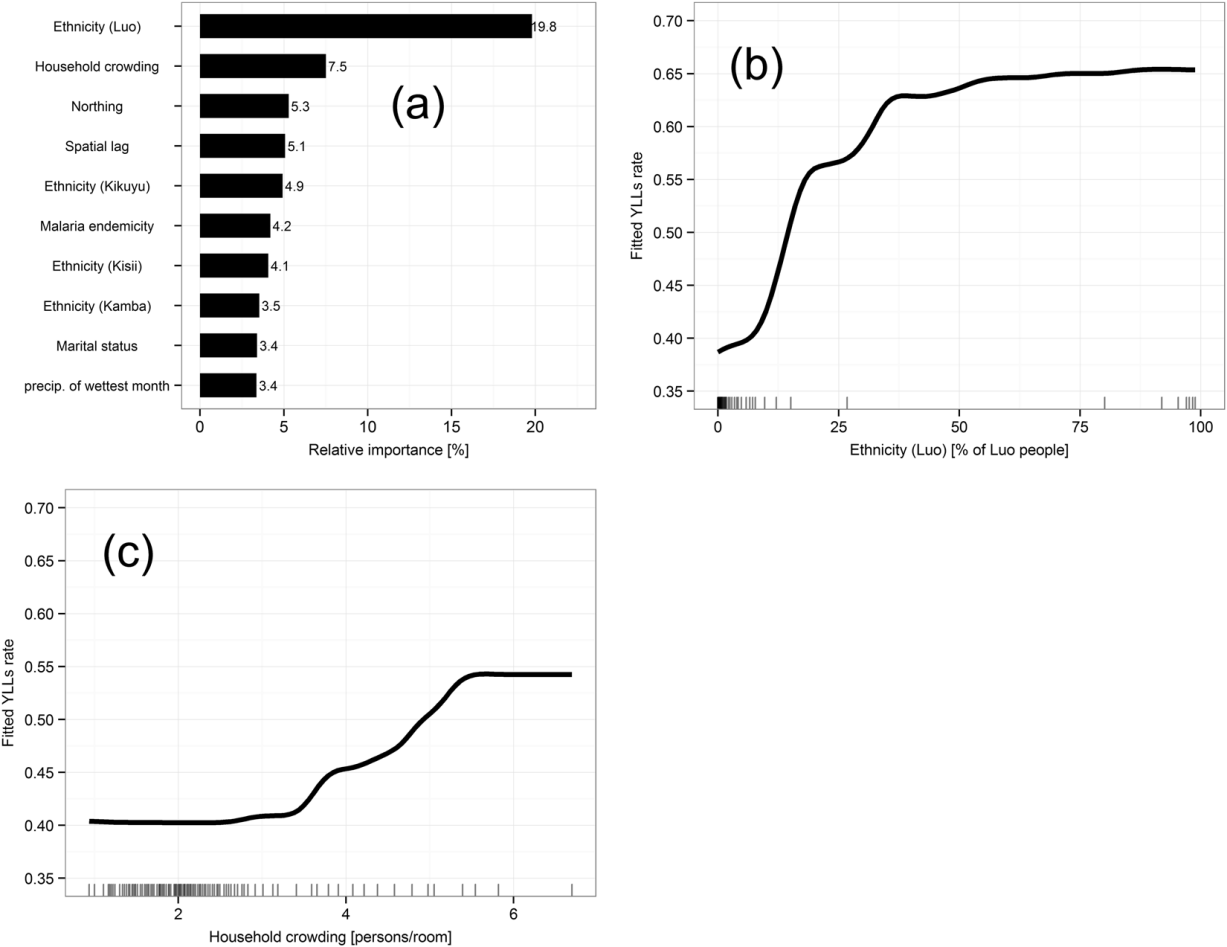
Explanatory variables associated with years of life lost (YLLs). Relative importance of the ten most influential variables (**a**) and partial dependence plots (PDPs) of the two most important variables: Ethnicity (Luo) (**b**) and household crowding (**c**). Rug plots on the x-axes illustrate the data distribution of the respective variable in percentiles. PDPs were smoothed using a spline interpolation.

**Figure 4:**
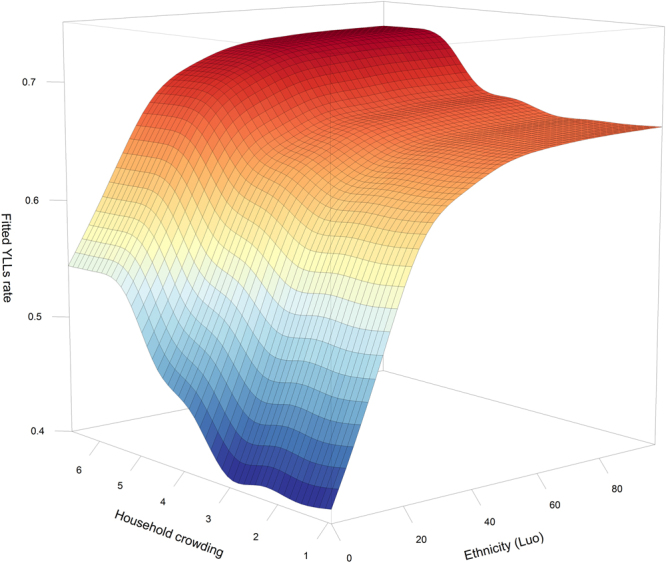
Joint partial dependence plot (PDP) visualizing interaction between ethnicity (Luo) and household crowding.

Figure 3(a) shows the relative importance of the significant explanatory variables in our model. Table 1 in the supplementary file shows odds ratios and 95% confidence intervals for the ten most important variables from a replicated version (Poisson multivariable regression) of our boosted regression tree (not reported). Higher shares of Luo ethnicity or more crowded households were strongest factors significantly and positively associated with YLL at the division level. Figure 3(b) and (c) depict the partial dependence plots (PDP) for share of Luo ethnicity and household crowding, each illustrating the isolated influence of these risk factors on YLLs while controlling for all other factors. For example, YLL sharply increased with higher share of Luo people until it levelled out at around 65%, after which the strength of association remained constant. Household crowding also had a non-linear influence on YLL. The effect of crowding on YLL was low for less than 3.5 persons per room but crowding above this threshold was associated with rapidly increasing YLLs rates, up to 5.5 persons per room. Shares of Luo ethnicity and crowded households in a division were also significantly interacting with each other (Fig. 4). The association between share of household crowding and YLL rate was stronger in divisions with a share of Luo people above approximately 30%.

**Table 1:** List of principle components used as explanatory variables in this study and the respective original variables with main factor loadings given as Pearson’s correlation coefficients in brackets

Principal component	Original variable and factor loading
Marital status	The share of people being married monogamously (0.8), or never being married (−0.6) were correlated with this component.
Protestant Christian	The share of people being Protestant (0.9), or Catholic (−0.3) were correlated with this component.
Occupation	The share of people working on family-owned farms (0.8) was correlated with this component.
Bicycle possession	The share of households (HH) having a bicycle (0.8).
Modern assets	The share of HH with a mobile (0.6), a TV (0.4) and a radio (0.3) were correlated with this component.
Livestock possession	The mean number of goats (0.7) and chicken (0.5) per HH were correlated with this component.
Poor cooking fuel	The share of HH cooking with firewood (0.9), or with charcoal (−0.5) were correlated with this component.
Poor lighting fuel	The share of HH using tin lamps (0.9) was correlated with this component.
Good roof material	The share of HH having an iron sheet roof (0.8), or a grass roof (−0.6) were correlated with this component.
Poor floor material	The share of HH having earth floor (0.7), or cement floor (−0.7) were correlated with this component.
Poor wall material	The share of HH having mud/wood as wall material (0.9) was correlated with this component.
Good sanitation	The share of HH having a covered pit latrine (0.9), or using the bush for sanitation (−0.4) were correlated with this component.
Poor water source	The share of HH using a river as water source (0.9) was correlated with this component.
Good water source	The share of HH using water drawn through pipes (0.7) was correlated with this component.

.

Furthermore, higher shares of Kisii ethnicity, higher malaria endemicity, and divisions at higher latitudes were significantly and positively associated with YLL (Fig. 3, Supplement). We also found that the spatial lag coefficient that represented YLL in neighboring divisions was positively associated with YLL. In contrast, higher shares of Kikuyu or Kamba ethnicity, higher shares of married people, or higher precipitation in divisions were significantly and negatively associated with YLL. We also tested our model with a spline function on the precipitation variable (precipitation of the wettest month) but could not find any significant difference to the model reported here (Supplementary File, Fig. 4).

## Discussion

Years of life lost due to premature mortality (YLL) were spatially clustered in western, northwestern, and northeastern Kenya and higher shares of Luo people and crowded households exhibited strongest associations with YLL in Kenyan divisions.

While most divisions displayed YLL rates around the national average of 0.4, some divisions had YLL rates up to four times higher (1.7), exhibiting spatial concentration of premature mortality. For example, high YLL rates clustered near Lake Victoria in the southwest (Fig. 2a). This region is characterized by highest HIV prevalence and high malaria endemicity^15^. HIV/AIDS and malaria are the first- and third-most important causes of YLL, constituting 18.9% and 10.0% of Kenya’s total YLL, respectively^4^. Hence, these conditions could be an explanation for the high burden in this area. Other significant clusters of high YLL rates were identified in Turkana County of northwestern Kenya (Fig. 2b) and in the border triangle with Ethiopia and Somalia (Fig. 2c). These predominantly remote, (semi-) arid regions are sparsely populated and dominated by (nomadic) pastoralism^16^. There could be several explanations for the high burden in these regions. First, remoteness could imply limited access to health care facilities and services. Second, low agricultural potential, combined with frequent droughts may periodically lead to health-threatening food insecurity^16^. Finally, inter-tribal violence related to resource scarcity and cross-border overflow from armed conflicts in neighboring countries (Somalia, South Sudan, Uganda) may be reasons for the clusters of high YLL in these regions^17,18^. The central and southern regions of Kenya, in which low YLL clustered, are rather characterized by higher agricultural potential, good income opportunities and better food security^16^. Combined with modest HIV prevalence and low malaria endemicity, this may explain the low burden in this area^15,19^.

Higher shares of specific ethnicities (Luo or Kisii) within divisions were positively associated with YLL and this association was the strongest among all variables in the model. Kenya is home to over 70 distinct ethnic groups, with the Kalenjin, Kamba, Kikuyu, Luo, and Luhya being among the largest ones. This rich diversity however has often led to social tensions^20,21^ and unequal health outcomes. For example, our finding is consistent with other studies that report highest HIV and tuberculosis prevalence and also child mortality among the Luo compared to other ethnicities in Kenya^22,23^. It is therefore quite understandable that those divisions inhabited primarily by the Luo or Kisii were positively associated with YLL. Our findings underline the importance of considering ethnicities when examining the burden of disease^24^. For example, certain health-related practices (e.g., circumcision, use of cultural medicine, sexual behavior) and people’s access to health care can be strongly dependent on ethnicity^24–26^. However, we here explicitly point out that we neither can assume a direct relationship between ethnicity and higher risk of YLL since we examined relationships at the ecological and not at the individual level. Nor can we infer causal relationships between ethnic-specific health behavior and YLL from our cross-sectional study. Future studies should look into the ethnic composition and respective health behavior at the individual level to better understand the burden of disease across different population groups and regions across Kenya.

Household crowding (over 3 persons per room) was positively associated with YLL, possibly due to a higher risk of communicable diseases such as acute respiratory infections, tuberculosis, or skin diseases with more persons sharing one room^27^. This finding is in line with studies from New Zealand^28^ and Uganda^29^ that also revealed associations between household crowding and morbidity. In contrast to our results, Ombok *et al*.^30^ did not identify crowding as a risk factor for child mortality in Nyanza Province of Kenya, possibly because they used a dichotomous variable (<5 and ≥5 persons/room) while we employed a continuous measure. There was a statistically significant interaction between household crowding and Luo ethnicity in our study. This indicates a mutually enforcing effect of these two factors so that risk of premature mortality is particular high in a division if both factors are high. While there is little evidence on the health effects of household crowding with respect to ethnicities in the literature, this suggests a need for more in-depth analysis in future studies.

We found higher malaria endemicity in a division was positively associated with YLL. This is consistent with a large body of literature, especially in the sub-Sahara Africa context^5,15,31–37^. In contrast, we found that being married can be protective against poor health and YLL; this has also been shown in several studies for different health outcomes^38–40^. Using the same data in another study at the individual level in Kenya, Gruebner *et al*.^40^ found reduced risk of child death for mothers who lived in households with married household heads. The authors assumed that being married indicates a stable living arrangement providing a health-promoting environment. In the current study, this may also be true at the ecological level as we found higher rates of married persons in a division was negatively associated with YLL.

Our study found a negative association of higher precipitation in a division with YLL. It is not entirely clear why this is the case. While one study found that malaria mortality was associated with rainfall in western Kenya^41^, a study in Sweden found that higher precipitation decreased the number of deaths in the 18^th^ and 19^th^ century^42^. The authors argue that in Sweden a warm spring with good rainfall increased the chance of a rich harvest, on which the pre-industrial population was dependent. This may also be true in our study, as precipitation allows for crop cultivation (e.g. coffee, banana) that would provide income possibilities for the local population with positive effects for health^43,44^.

Divisions that were geographically located further in the north of Kenya were positively associated with YLL. This may mirror findings from our spatial cluster analysis suggesting that these regions may represent remoteness, low agricultural potential, frequent droughts, or inter-tribal violence. More spatial epidemiological studies are needed to further breakdown the geographic distribution of explanatory variables associated with the burden of disease in Kenya.

Furthermore, YLL were positively associated with YLL in neighboring divisions. This may indicate spill over, that is, exposure factors in one division (e.g., higher share of specific ethnicities, crowded households, malaria endemicity) may also be associated with higher YLL in adjacent divisions, even when these factors are low there. Another explanation for the spatial lag effect could be that adjacent divisions share similar high values of exposure factors.

We recognize three noteworthy limitations of our study. First, we calculated rates of YLL based on death cases per household within the last twelve months prior to the census that can be related to possible biases. For example, early death of a child is a traumatic event that may influence such reporting. Recall bias may play a role due to exclusion of deaths that occurred within the recall period and may underestimate the level of mortality. In turn, over-reporting of deaths that occurred outside the recall period may have led to an overestimation of mortality^45^. Although recall bias has frequently been regarded as a major issue in case-control studies, it has also been reported to compromise retrospective study designs^46^. For the neighboring country of Tanzania however, Moshiro *et al*.^47^ found that long recall periods of up to 12 months did not affect estimates.

Second, we had to exclude 9.6% of the death cases as they were reported with an unknown age at death. Comparisons between age-specific mortality rates calculated from the Kenyan census data with rates from the GBD 2010 study indicated noticeable lower mortality rates for older ages (>60) in our data. This suggests that the death cases that we excluded in our study were predominantly people of older age. Death cases at older age have a fairly small impact on the YLL due to lower residual life expectancies and hence we assume that it had only a negligible effect on our findings.

Third, we created a single model for YLL attributable to all causes of deaths based on census, that is, on a complete enumeration of the population. Such an approach prevents analysis of YLL specific to communicable diseases, non-communicable diseases, or injuries. Yet, risk factors vary substantially from one group of diseases to another, which needs to be kept in mind when interpreting our findings.

To the best of our knowledge this is the first study that addressed the spatial distribution of the burden of disease due to premature mortality at the division level in Africa. Based on census data, we identified spatial patterns of the years of life lost (YLL) that provide crucial information for better understanding about the locations where people are at higher risk for premature mortality. Moreover, we identified exposure factors that were significantly associated with YLL.

Kenya has made significant improvements in the reduction of the top three causes of premature death in 2016 as compared to 2005^48^. For example, HIV/AIDS as a cause for premature death was reduced by 60.4%, diarrheal diseases by 29.8%, and lower respiratory infections by 23.3%^48^. Furthermore, Malaria as the seventh important cause of premature death in the country was reduced by 59.9% as compared to 2005^48^.

Our spatial epidemiological approach with census data is transferable and should be reapplied with updated census data once these are available. Thereby it will contribute to a precision public health supporting the allocation of scarce resources to regions and specific populations most affected by premature mortality also in contexts beyond Kenya.

## Methods

### Data set and availability

Micro level data from the most recent Census conducted August 24^th^ 2009^49^ was used. This data is also available to other researchers who meet the criteria for access to confidential information. Interested researchers may request this data at datarequest@knbs.or.ke.

### Study design and population

As in Gruebner *et al*.^40^, a cross-sectional study design was used, with data on the general population and for this study aggregated at the division level. We excluded those divisions with preliminary non-residential areas and thereby arrived at N = 612 divisions suitable for our analyses. The population for these divisions ranged from 165 to 870,202, with a median population of 44,661.

### Outcome variable

The outcome variable was “Years of Life Lost (YLL)” per person at the division level, calculated based on reported death cases in each household 12 months prior to the census, and standardized by age and gender. YLLs are defined as the sum of years of residual life expectancy of each death case with regard to the GBD 2010 standard life table that assumes a life expectancy at birth of 86.02 years for all individuals globally^1^. The census reports 263,564 death cases in Kenya, however, with 9.7% of them recorded with an unknown age of the deceased person. These cases were excluded from our study since they could not be used for calculating YLLs. Our final dataset included 238,121 death cases that were used to calculate age and sex standardized YLL rates at the division level (N = 612).

### Explanatory variables

We considered the following variables from the census aggregated at the division level: Population density (population/km^2^), household crowding (mean number of persons/room), percentage of rural households and ethnic population groups, as well as mean educational attainment (range 0 = no education to 20 = completed university degree).

Mean access to health care was calculated based on health facilities obtained from the Kenya Open Data Portal^50^ to population ratio. Malaria endemicity (i.e., basic reproductive number for Malaria cases) was taken from Gething *et al*.^15^ and the mean altitude in meter was taken from Jarvis *et al*.^51^. Six variables represented climate related factors and were taken from Hijmans *et al*.^52^: Mean annual temperature in degrees centigrade with maximum temperature of warmest month and minimum temperature of coldest month, as well as the mean annual precipitation in millimeter with mean precipitation of wettest month and mean precipitation of driest month. We also included geographic coordinates and a factor representing the spatial lag of YLL (i.e., average value of YLL in adjacent divisions).

Furthermore, we applied a principal components analysis on additional census variables to combine explanatory variables representing socio-demographic characteristics of the population to enhance the interpretability of results^53,54^. All components with Eigenvalues greater than one were extracted and used as uncorrelated explanatory factors in our analyses. Table 1 summarizes all principal components with respective variables and factor loadings and Table 2 provides summary statistics for all variables used in the analysis.

**Table 2:** Descriptive statistics for all explanatory variables used in the study.

		Mean	SD	Median	Min	Max
Outcome	Years of life los (YLL)	0.42	0.21	0.36	0.00	1.66
Demographic variables	Marital status (PC)	0.19	7.80	−0.33	−20.28	34.51
	Protestant Christian (PC)	−7.24	22.14	−4.40	−59.36	46.76
	Population/km^2^	447	1,538	185	0	23,36
	Mean number of persons per room	2.38	1.09	2.08	0.94	6.70
	% Rural households	81.16	30.31	100.00	0.00	100
	% Ethnicity (Kamba)	11.83	29.71	0.19	0.00	99.66
	% Ethnicity (Kikuyu)	12.34	27.86	0.24	0.00	99.03
	% Ethnicity (Kisii)	4.17	17.66	0.12	0.00	99.22
	% Ethnicity (Luo)	7.54	23.17	0.23	0.00	98.87
	% Ethnicity (Luhya)	9.32	24.57	0.38	0.00	98.78
	% Ethnicity (Kalenjin)	14.56	31.63	0.23	0.00	99.58
	% Ethnicity (Somali)	12.06	31.05	0.14	0.00	99.91
	% Ethnicity (Other)	28.18	39.42	2.92	0.01	99.90
Socio-economic variables	Occupation (PC)	0.84	16.91	0.02	−31.34	55.10
	Bicycle possession (PC)	−3.72	26.05	1.25	−63.90	47.00
	Modern assets (PC)	−1.90	21.42	−2.55	−47.48	58.13
	Livestock possession (PC)	5.30	15.79	0.28	−5.96	152.08
	Poor cooking fuel (PC)	−10.71	25.80	−0.20	−106.04	11.48
	Poor lighting fuel (PC)	−4.49	26.36	−8.23	−56.23	55.39
	Good roof material (PC)	−16.59	38.88	−3.39	−110.83	26.53
	Poor floor material (PC)	−8.43	31.71	−0.14	−107.79	31.36
	Poor wall material (PC)	−12.52	31.77	−11.50	−65.29	44.17
	Good sanitation (PC)	−12.06	34.06	−3.97	−78.41	44.24
	Poor water source (PC)	−3.70	23.15	−5.77	−39.72	59.32
	Good water source (PC)	−0.10	20.25	−3.31	−37.79	67.18
	Mean educational attainment	4.70	1.98	5.13	0.12	10.18
	Mean access to health care	1.92	7.52	1.31	0.00	181.82
Environmental variables	Mean malaria endemicity	3.41	10.73	1.00	0.00	91.30
	Mean altitude in meter	1,287	661	1,355	7	2,928
	Annual mean temperature in degree centigrade	210.9	37.29	210.0	123.0	291
	Maximum temperature of warmest month in degree centigrade	301.8	35.6	301.0	208.0	400
	Minimum temperature of coldest month in degree centigrade	129.2	41.13	123.0	47.0	222
	Annual mean precipitation in millimeter	1031.9	448.7	978.0	186.0	2441.0
	Precipitation of wettest month in millimeter	198.9	73.4	198.0	52.0	627
	Precipitation of driest month in millimeter	25.9	23.2	21.0	0	87
	Longitude	36.79	36.39	36.70	33.99	41.83
	Latitude	−0.28	1.64	−0.32	−4.49	5.13
	Spatially lagged YLL	0.41	0.15	0.42	0.10	0.70

The level of analysis were the divisions (N = 612).

### Analysis

We first performed spatial autocorrelation analysis (Moran’s I) to explore spatial clustering of YLL, that is, the degree to which nearby divisions tend to show similar or dissimilar YLLs rates. The *global* Moran’s I characterizes the overall pattern in the entire study area^55^. The *local* Moran’s I identifies local spatial clusters of similar (hotspots) or dissimilar neighboring divisions (outliers) that are significantly different from an expected spatial pattern under normality assumption^56^. Divisions that indicated a significant (p < 0.001) local Moran’s I were mapped and classified into High-High (or Low-Low) hotspots, that is, high (or low) YLL in one division next to high (or low) YLL in neighboring divisions, or Low-High or High-Low spatial outliers. We conducted global and local Moran’s I with “spdep” in R^57,58^.

Second, we used boosted regression trees (BRTs) to quantify the association between explanatory variables and YLL in Kenya. BRTs draw on techniques from machine learning^59,60^ and have been successfully applied to disease modeling^60–63^. We chose BRTs because they can handle non-linear relationships, are insensitive to outliers, and account for interactions between variables^60,64^. Generally, models based on regression trees partition the variable space into those parts with the most homogenous responses to the explanatory variables^60,64^ and the relative importance of these variables determines the strength of their association with the YLL. This relative importance is quantified by the number of times a variable is used for splitting a regression tree, weighted by the model improvements as a result of each additional split, and averaged over all trees^60,64^. In order to examine the nature of the association between a variable and YLL, partial dependence plots (PDPs) were computed. PDPs are fitted functions for a certain explanatory variable along its data range and thus represent the isolated effect of the variable on YLL while holding all other explanatory variables at their mean^60^. Interactions among variables identified and modeled by BRTs can be visualized by three-dimensional PDPs. We applied BRTs using “dismo” and “gbm” packages in R^58,65,66^.

Finally, we tested BRT model residuals for spatial autocorrelation to verify the assumption of independent errors^67^. For all procedures, we followed the guidelines and recommendations of Good Epidemiological Practice (GEP) defined by the German Society for Epidemiology to secure ethical principals in data handling^68^.

## Electronic supplementary material

Supplementary file

## References

[1] Murray CJL (2012). Disability-adjusted life years (DALYs) for 291 diseases and injuries in 21 regions, 1990–2010: a systematic analysis for the Global Burden of Disease Study 2010. Lancet.

[2] Horton R (2012). GBD 2010: understanding disease, injury, and risk. Lancet.

[3] Murray CJL (2012). GBD 2010: design, definitions, and metrics. Lancet.

[4] Institute for Health Metrics and Evaluation. GBD Data Visualizations. at, http://www.healthdata.org/gbd/data-visualizations (2017).

[5] Wachira B, Martin IBK (2011). The state of emergency care in the Republic of Kenya. African. J. Emerg. Med..

[6] Polinder S, Haagsma JA, Stein C, Havelaar AH (2012). Systematic review of general burden of disease studies using disability-adjusted life years. Popul. Health Metr..

[7] *The Global Burden of Infectious Diseases*. 3–21, 10.1007/978-0-387-93835-6_1 (Springer New York, 2010).

[8] Dodhia H, Phillips K (2008). Measuring burden of disease in two inner London boroughs using Disability Adjusted Life Years. J. Public Health (Bangkok)..

[9] Wachira, L. J. M., Muthuri, S. K., Tremblay, M. S. & Onywera, V. O. Results from Kenya’s 2014 Report Card on the Physical Activity and Body Weights of Children and Youth. *J. Phys. Act. Heal*. at, http://www.ku.ac.ke/schools/human_sciences/images/stories/research/Results-From-Kenyas-2014-Report-Card-on-the-Physical-Activity-and-body-weight-of-children-and-youth.pdf (2014). 10.1123/jpah.2014-0169 25426917

[10] Mariotti S, D’Errigo P, Mastroeni S, Freeman K (2002). Years of life lost due to premature mortality in Italy. Eur. J. Epidemiol..

[11] Zhou S-C (2011). Measuring the burden of disease using disability-adjusted life years in Shilin County of Yunnan Province, China. Environ. Health Prev. Med..

[12] MacNab YC (2007). Mapping disability-adjusted life years: a Bayesian hierarchical model framework for burden of disease and injury assessment. Stat. Med..

[13] MacNab YC (2009). Bayesian multivariate disease mapping and ecological regression with errors in covariates: Bayesian estimation of DALYs and ‘preventable’ DALYs. Stat. Med..

[14] Manda SOM, Abdelatif N (2017). Smoothed Temporal Atlases of Age-Gender All-Cause Mortality in South Africa. Int. J. Environ. Res. Public Health.

[15] Gething PW (2011). A new world malaria map: Plasmodium falciparum endemicity in 2010. Malar. J..

[16] Mwagore, D. *Land Use in Kenya: The case for a national landuse policy*. at, http://scholar.google.com/scholar?q=related:_TW9jMsXRkUJ:scholar.google.com/&amp;hl=en&amp;num=20&amp;as_sdt=0,5 (Kenya Land Alliance, 2003).

[17] Kumssa A, Jones JF (2009). & Herbert Williams. J. Conflict and human security in the North Rift and North Eastern Kenya. Int. J. Soc. Econ..

[18] Leff J (2009). Pastoralists at War: Violence and Security in the Kenya-Sudan-Uganda Border Region. Int. J. Confl. Violence.

[19] Wamai, R. G. The Kenya Health System—Analysis of the situation and enduring challenges. *Jmaj* at, http://www.med.or.jp/english/pdf/2009_02/134_140.pdf (2009).

[20] Kasara K (2013). Separate and Suspicious: Local Social and Political Context and Ethnic Tolerance in Kenya. J. Polit..

[21] African Studies Center. Kenya - Ethnic Groups. *University of Pennsylvania, East Africa Living Encyclopedia* (1998).

[22] Odhiambo FO (2012). Profile: The KEMRI/CDC Health and Demographic Surveillance System–Western Kenya. Int. J. Epidemiol..

[23] Shaffer DN (2010). HIV-1 Incidence Rates and Risk Factors in Agricultural Workers and Dependents in Rural Kenya: 36-Month Follow-Up of the Kericho HIV Cohort Study. JAIDS. J. Acquir. Immune Defic. Syndr..

[24] Eshetu, E. B. & Woldesenbet, S. A. Are there particular social determinants of health for the world’s poorest countries? *Afr. Health Sci*. **11** (2011). PMC3092326 21572866

[25] World Health Organization. *Closing the Gap in a Generation*. at, http://books.google.de/books?id=zc_VfH7wfV8C&amp;pg=A32&amp;dq=intitle:Closing+the+gap+in+a+generation&amp;hl=&amp;cd=1&amp;source=gbs_api (World Health Organization, 2008).

[26] Marmot M (2008). Closing the gap in a generation: health equity through action on the social determinants of health. Lancet.

[27] Adler NE, Newman K (2002). Socioeconomic disparities in health: pathways and policies. Health Aff. (Millwood)..

[28] Baker M, Das D, Venugopal K, Howden-Chapman P (2008). Tuberculosis associated with household crowding in a developed country. J. Epidemiol. Community Heal..

[29] Herrin WE, Amaral MM, Balihuta AM (2013). The relationships between housing quality and occupant health in Uganda. Soc. Sci. Med..

[30] Ombok M (2010). Geospatial distribution and determinants of child mortality in rural western Kenya 2002–2005. Trop. Med. &amp; Int. Heal..

[31] Selemani M (2015). Spatial and space-time clustering of mortality due to malaria in rural Tanzania: evidence from Ifakara and Rufiji Health and Demographic Surveillance System sites. Malar. J..

[32] Rumisha SF, Smith TA, Masanja H, Abdulla S, Vounatsou P (2014). Relationship between child survival and malaria transmission: an analysis of the malaria transmission intensity and mortality burden across Africa (MTIMBA) project data in Rufiji demographic surveillance system, Tanzania. Malar. J..

[33] Caminade C (2014). Impact of climate change on global malaria distribution. Proc. Natl. Acad. Sci..

[34] Gething PW (2012). A Long Neglected World Malaria Map: Plasmodium vivax Endemicity in 2010. PLoS Negl. Trop. Dis..

[35] Kimani-Murage EW (2014). Trends in childhood mortality in Kenya: The urban advantage has seemingly been wiped out. Health Place.

[36] Kleinschmidt I (2009). Marked increase in child survival after four years of intensive malaria control. Am. J. Trop. Med. Hyg..

[37] Schellenberg JA (1998). An analysis of the geographical distribution of severe malaria in children in Kilifi District, Kenya. Int. J. Epidemiol..

[38] Smith-Greenaway E, Trinitapoli J (2014). Polygynous Contexts, Family Structure, and Infant Mortality in Sub-Saharan Africa. Demography.

[39] Balayla J, Azoulay L, Abenhaim HA (2011). Maternal Marital Status and the Risk of Stillbirth and Infant Death: A Population-Based Cohort Study on 40 Million Births in the United States. Women’s Heal. Issues.

[40] Gruebner O (2015). Place of Residence Moderates the Risk of Infant Death in Kenya: Evidence from the Most Recent Census 2009. Plos One.

[41] Sewe M (2015). The association of weather variability and under five malaria mortality in KEMRI/CDC HDSS in Western Kenya 2003 to 2008: a time series analysis. Int. J. Environ. Res. Public Health.

[42] Schumann B, Edvinsson S, Evengård B, Rocklöv J (2013). The influence of seasonal climate variability on mortality in pre-industrial Sweden. Glob. Health Action.

[43] Gruebner, O. *et al*. Urban health in megacities: Extending the framework for developing countries. *Int. Hum. Dimens. Program*. at, http://www.bonn.unu.edu/file/get/7923.pdf#page=44 (2011).

[44] Galea S, Freudenberg N, Vlahov D (2005). Cities and population health. Soc. Sci. Med..

[45] Depoortere E (2004). Violence and mortality in West Darfur, Sudan (2003–04): epidemiological evidence from four surveys. Lancet.

[46] Hassan, E. *Recall bias can be a threat to retrospective and prospective research designs*. at, http://scholar.google.com/scholar?q=related:gCX2H6FpGvcJ:scholar.google.com/&amp;hl=en&amp;num=20&amp;as_sdt=0,5 (The Internet Journal of Epidemiology, 2006).

[47] Moshiro C (2005). Effect of recall on estimation of non-fatal injury rates: a community based study in Tanzania. Inj. Prev..

[48] Institute for Health Metrics and Evaluation. What causes the most premature death? *Kenya country profile* at, http://www.healthdata.org/kenya (2018).

[49] Statistics, K. K. N. B. of. *Kenya National Population and Housing Census* 2009. at, http://www.knbs.or.ke/index.php?option=com_phocadownload&amp;view=category&amp;id=109:population-and-housing-census-2009&amp;Itemid=599# (KNBS, 2010).

[50] Kenya, I. C. T. Board. Opendata.go.ke. at, http://www.opendata.go.ke/.

[51] Jarvis, A., Reuter, H. I., Nelson, A. & Guevara, E. Jarvis: Hole-filled SRTM for the globe version 4, 2008. at, http://srtm.csi.cgiar.org (2014).

[52] Hijmans, R. J., Cameron, S. E. & Parra, J. L. Very high resolution interpolated climate surfaces for global land areas. *Int. J*. … at, http://bio.research.ucsc.edu/~barrylab/classes/climate_change/HijmansIJC2005.pdf (2005).

[53] Jolliffe, I. T. *Principal component analysis*. at, http://books.google.de/books?id=_olByCrhjwIC (Springer, 2002).

[54] Vyas S, Kumaranayake L (2006). Constructing socio-economic status indices: how to use principal components analysis. Health Policy Plan..

[55] Bivand, R. S., Pebesma, E. & Gómez-Rubio, V. Applied Spatial Data Analysis with R. at, http://books.google.com/books?id=v0eIU9ObJXgC&amp;pg=PA14&amp;dq=intitle:Applied+Spatial+Data+Analysis+with+R&amp;hl=&amp;cd=1&amp;source=gbs_api (Springer Science - Business Media, 2013).

[56] Anselin L (1995). Local Indicators of Spatial Association-LISA. Geogr. Anal..

[57] Bivand, R. S. *et al*. Spdep: Spatial dependence: weighting schemes, statistics and models. R package version 0.4-34. at, http://cran.r-project.org/web/packages/spdep/index.html (2009).

[58] Team, R. D. C. *R: A language and environment for statistical computing*. at, http://www.r-project.org (R Foundation for Statistical Computing, 2013).

[59] Breiman L (2001). Statistical Modeling: The Two Cultures (with comments and a rejoinder by the author). Stat. Sci..

[60] Elith J, Leathwick JR, Hastie T (2008). A working guide to boosted regression trees. J. Anim. Ecol..

[61] Cheong YL, Leitão PJ, Lakes T (2014). Assessment of land use factors associated with dengue cases in Malaysia using Boosted Regression Trees. Spat. Spatiotemporal. Epidemiol..

[62] Stevens KB, Pfeiffer DU (2011). Spatial modelling of disease using data- and knowledge-driven approaches. Spat. Spatiotemporal. Epidemiol..

[63] Bhatt S (2013). The global distribution and burden of dengue. Nature.

[64] Hastie, T., Tibshirani, R. & Friedman, J. *The Elements of Statistical Learning*. at, http://books.google.de/books?id=yPfZBwAAQBAJ&amp;printsec=frontcover&amp;dq=intitle:The+Elements+of+Statistical+Learning+Data+Mining+Inference+Trevor+Hastie+Springer&amp;hl=&amp;cd=1&amp;source=gbs_api (Springer Science - Business Media, 2013).

[65] Hijmans, R. J., Phillips, S., Leathwick, J. & Elith, J. Package &apos;dismo&apos; Circles at, ftp://sourceforge.c3sl.ufpr.br/CRAN/web/packages/dismo/dismo.pdf (2016).

[66] Ridgeway, G., Southworth, M. H. & RUnit, S. Package &apos;gbm&apos; Viitattu at, http://citeseerx.ist.psu.edu/viewdoc/download?doi=10.1.1.398.7110&amp;rep=rep1&amp;type=pdf (2013).

[67] Crase B, Liedloff AC, Wintle BA (2012). A new method for dealing with residual spatial autocorrelation in species distribution models. Ecography (Cop.).

[68] Hoffmann W, Latza U, Terschüren C (2005). Guidelines and Recommendations for Ensuring Good Epidemiological Practice (GEP) - Revised Version after Evaluation. Das Gesundheitswes..

