# Supplementary material for: Modeling and mapping the burden of disease in Kenya
Source: Sci Rep. 2018 Jun 29;8:9826. doi: 10.1038/s41598-018-28266-4 (PMC6026135; doi:10.1038/s41598-018-28266-4)
Supplement: Supplementary file 1 — Supplementary file [file 41598_2018_28266_MOESM1_ESM.docx]

Supplementary file

Title: Modeling and mapping the burden of disease in Kenya

Michael Frings, Tobia Lakes, Daniel Müller, MMH Khan, Michael Epprecht, Samuel Kipruto, Sandro Galea, Oliver Gruebner

| **Variable** | **Odds Ratio** | **95% Confident Interval** |
| --- | --- | --- |
| Ethnicity (Luo) | 1.007 | 1.007-1.007 |
| Household crowding | 1.068 | 1.059-1.077 |
| Northing | 1.000 | 1.000-1.000 |
| Spatial lag | 1.183 | 1.133-1.234 |
| Ethnicity (Kikuyu) | 1.002 | 1.002-1.002 |
| Malaria endemicity | 1.003 | 1.003-1.004 |
| Ethnicity (Kisii) | 1.002 | 1.002-1.002 |
| Ethnicity (Kamba) | 1.002 | 1.001-1.002 |
| Marital status (yes ) | 0.993 | 0.993-0.994 |
| Precipitation of wettest month | 1.000 | 1.000-1.000 |

Table 1: Multivariable Poisson regression on the 10 most important variables found in the boosted regression tree model.


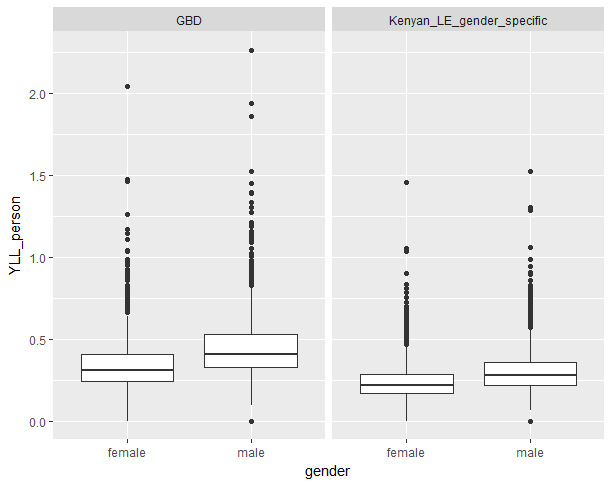


Figure 1: Comparison between years of life lost (YLL) per person for the global (86 years) versus the Kenyan specific (54 years) life expectancy across gender.


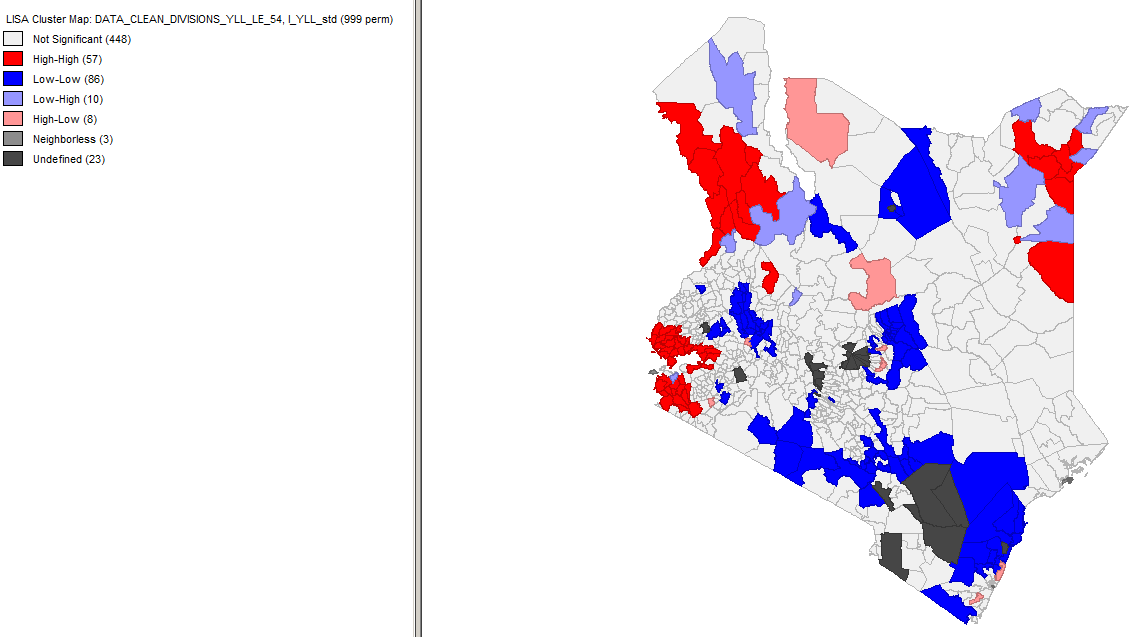

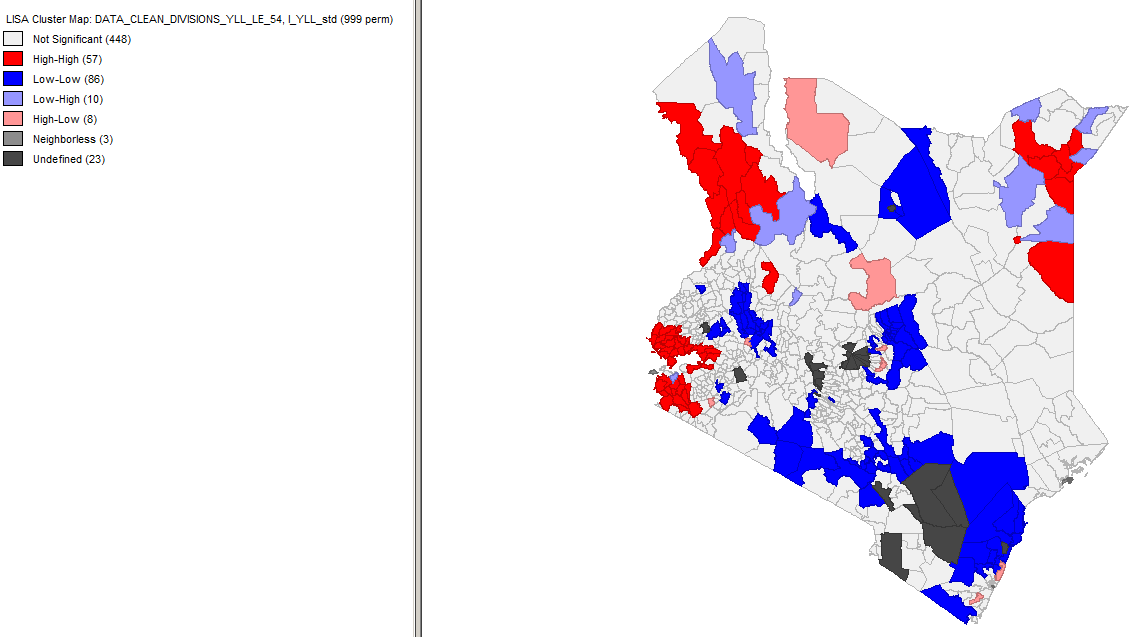


Figure 2: Significant spatial clusters of years of life lost (YLLs) per person at the division level for the Kenyan specific life expectancy of 54 years. Similar to the global life expectancy of 86 years that was used in the main text, the map shows three clusters of divisions in which high values of YLL (above average) were found next to each other, one near Lake Victoria, one in Turkana County, and one in the border triangle with Ethiopia and Somalia.





Figure 3: Partial dependence plots (PDPs) of the remaining variables of Figure 3 in the main document. Rug plots on the x-axes illustrate the data distribution of the respective variable in percentiles. PDPs were smoothed using a spline interpolation.


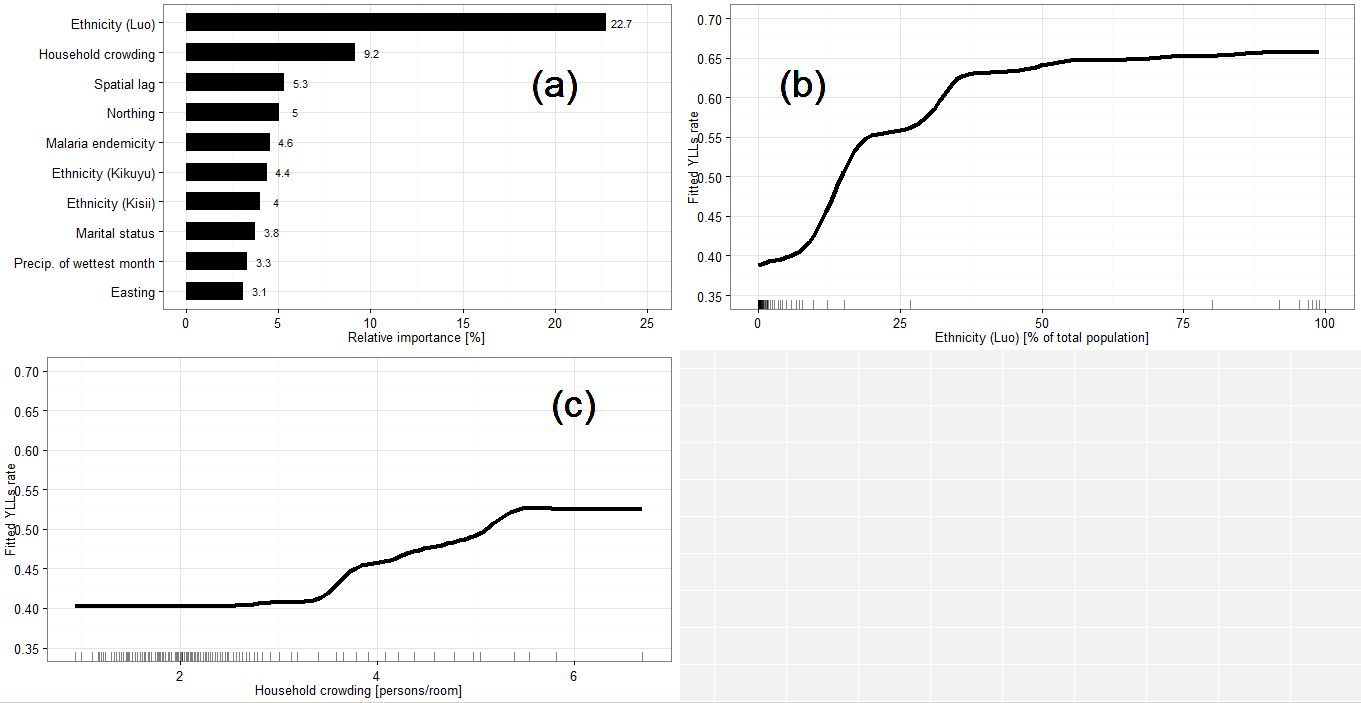


Figure 4: Modelled outcomes with the spline function on the variable “precipitation of the wettest month”. Rug plots on the x-axes illustrate the data distribution of the respective variable in percentiles. Small differences to the original Figure 3 in the main text are due to the bagging-fraction (randomness) in the boosted regression tree. We only show the 10 most important predictors of a total of 38 predictors. The small differences in the top 10 predictors are further due to the random component in the tree building process.
